# From mouthpiece of an emerging specialty to voice for high-quality research: the first 100 years of the *British Journal of Anaesthesia*

**DOI:** 10.1016/j.bja.2023.04.007

**Published:** 2023-05-15

**Authors:** Eleanor Shaw, Stephanie Snow, Carsten Timmermann

**Affiliations:** Centre for the History of Science, Technology and Medicine, University of Manchester, Manchester, UK

**Keywords:** academic medicine, anaesthesia research, history of anaesthesia, medical journal, National Health Service, publishing, Royal College of Anaesthetists

## Abstract

The *British Journal of Anaesthesia* (*BJA*) celebrates its centenary in 2023, and with it 100 yr of continuous anaesthesia research publication. As an editorially and financially independent journal, the *BJA* faced a rapidly changing anaesthesia profession, health system, and publishing world without the security of institutional support. In its early days, the Journal was vocal about the challenging conditions faced by anaesthetists before the National Health System was established, and was essential in advocating for the specialty. Although the years after World War II brought improving fortunes for the specialty, the *BJA* found itself struggling to publish. As the Journal's fortunes began to improve, a new research and healthcare context emerged, radically changing the face of anaesthesia research and practice, to which the Journal needed to adapt. In spite of a range of challenges throughout the years, the *BJA* has developed into an international, future-focused, well-respected publication. This could not have been achieved without continual transformation, and the willingness to take risks and meet the changing times head on.

**Editor's key points**

• As one of the oldest anaesthesia journals covering clinical and laboratory studies in anaesthesia, pain, and critical care medicine, the *British Journal of Anaesthesia* has played a major role in the development of the specialty.
• Early economic struggles were overcome as the Journal's role evolved to a leading journal and supporter of research and education in the field with global reach.
• Early adoption of innovations in medical and scientific publishing, including internationalisation of the board, use of electronic peer review, introduction of electronic publication, and use of open access publication positioned the *British Journal of Anaesthesia* to confront the continuing challenges facing medicine and anaesthesia over the past century.

At the time of the founding of the *British Journal of Anaesthesia* (*BJA*) in 1923, there were very few specialist anaesthetists, and the anaesthetic agents commonly on offer, ether, chloroform, and nitrous oxide, were used primarily by general practitioners. Figure 1 shows an image of an improved ether mask from the October 1923 issue of the *BJA.* The establishment of the *BJA* was an attempt to create for anaesthetists an organisation that could act ‘not only as the mouthpiece for those who desire to give public expression to the results of their research and experience, but to place before readers an account of what is being done generally in the anaesthetic world’.^1^ Over the next 100 yr, the *BJA* changed hugely. In the 1920s it was pulled together on a shoestring budget, without institutional backing and entering a publishing arena where few medical journals lasted the distance. Now it is an international entity, marking its success by impact factor and yearly revenue, and contributing to anaesthesia's research landscape. These changes reflect not only the significant change in the status and practice of anaesthesia as a specialty, but also the changing face and expectations of medical journals in the 20th century, the creation and ongoing evolution of the UK National Health Service, and the wider increase of biomedicine in this time period. As the *BJA* has moved away from its humble origins, the ideas that drive it and its mission have moved on too.

## Scientific and medical publishing

Established in 1923, the *BJA* was the second anaesthesia journal in the world, only behind the American journal *Current Researches in Anesthesia and Analgesia* (later *Anesthesia & Analgesia*), which was founded in 1922. In its aim to document the progress of a medical specialty, however, it was far from unique. Beginning with the foundation of the very first scholarly journals, the French *Journal des Sçavans* and the English *Philosophical Transactions* in 1665, there is a long and rich history of scientific and medical publication spanning more than 350 yr.^2^ In the late 19th and early 20th centuries, an expansion of the university sector resulted in an increase in both researchers and their research in science and medicine. As a result, the volume of research being submitted to the very active community of scientific journals in the UK increased. It was into this vibrant publishing sphere that the *BJA* entered with its first issue. The establishment of the *BJA* offered a way for the burgeoning specialty of anaesthesia to signal its unique scope of practice and independence, and it immediately set to work arguing for the establishment of a ‘British Association of Anaesthetists’ that could ‘advance the science and practice of anaesthesia’.^1^

**Fig 1:**
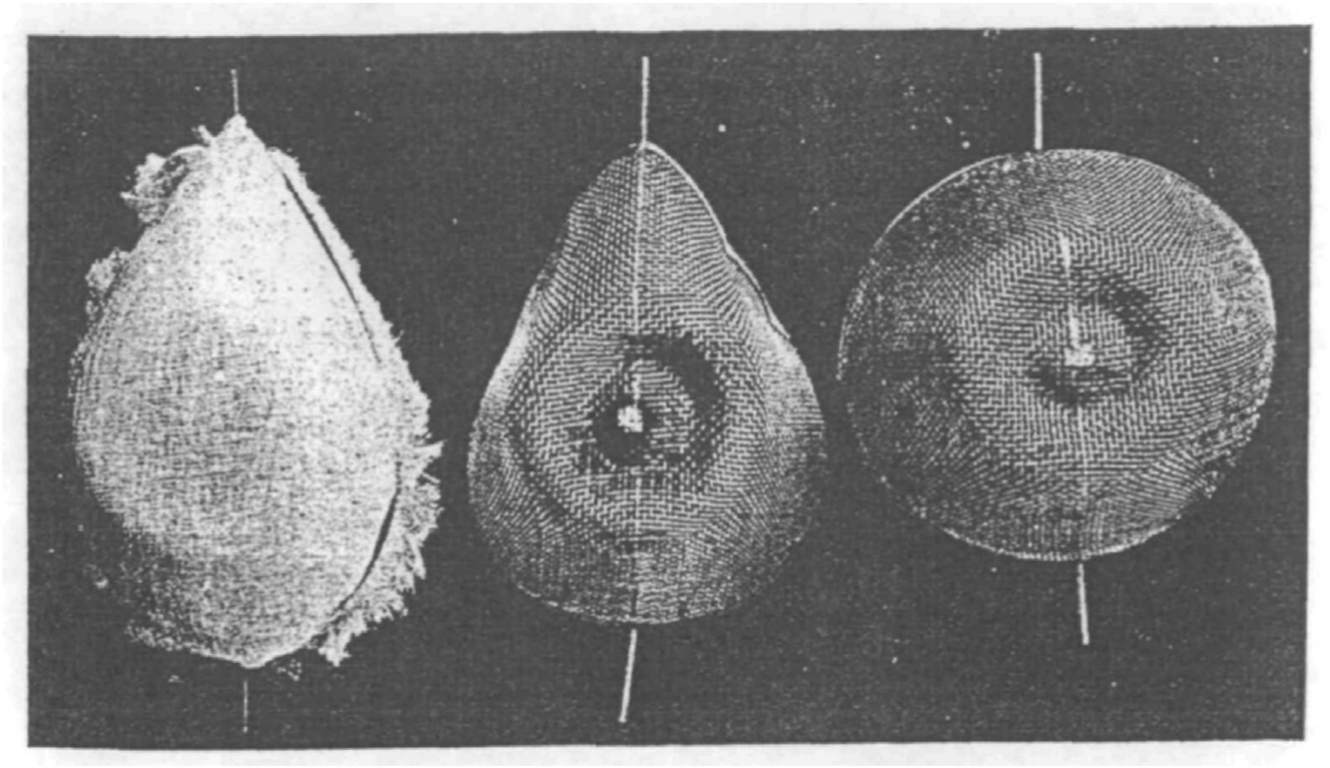
An improved ether mask proposed by S.R. Wilson. Reproduced with permission from *Br J Anaesth* 1923; **1**: 2.

## The beginnings of the *BJA*

Unlike many of its fellow medical journals, the *BJA* was an independent concern, not attached to any association, college, or society. Instead it worked with publisher and printer John Sherratt and Son of Manchester to produce the journal, reliant on subscription fees and advertisement revenue to fund its publication. Especially in the early days of the specialty, before the foundation of either the Association of Anaesthetists of Great Britain and Ireland or the Faculty of Anaesthetists of the Royal College of Surgeons of England, the journal was an essential mouthpiece for the concerns and aims of anaesthetists, regularly decrying the appalling pay and working conditions of the many of the specialty.^3^, ^4^, ^5^ Amongst the contributors to the first issues and the boards of the journal were the great and the good of 1920s anaesthesia, including D'Arcy Power, C. Langton Hewer, and Dudley Wilmot Buxton, bringing together their collective weight to support the endeavour.^6^ For further information on the early years of the journal, see Fitch and colleagues, for example.^6^

*‘It may or may not be good business on the part of lay boards to pay anaesthetists inadequate fees but on the question of general policy it is surely not in the interest of the public or the dignity of the medical profession that the services of qualified men and women, should be assessed at so low a value. That men and women can be found to undertake service which is so poorly rewarded is not sufficient warrant for a perpetuation of that grade of compensation.’*

‘Our hospital emoluments.’ *Br J Anaesth* 1928; **5**: p. 8. https://doi.org/10.1093/bja/5.3.107.^3^

**Fig 2:**
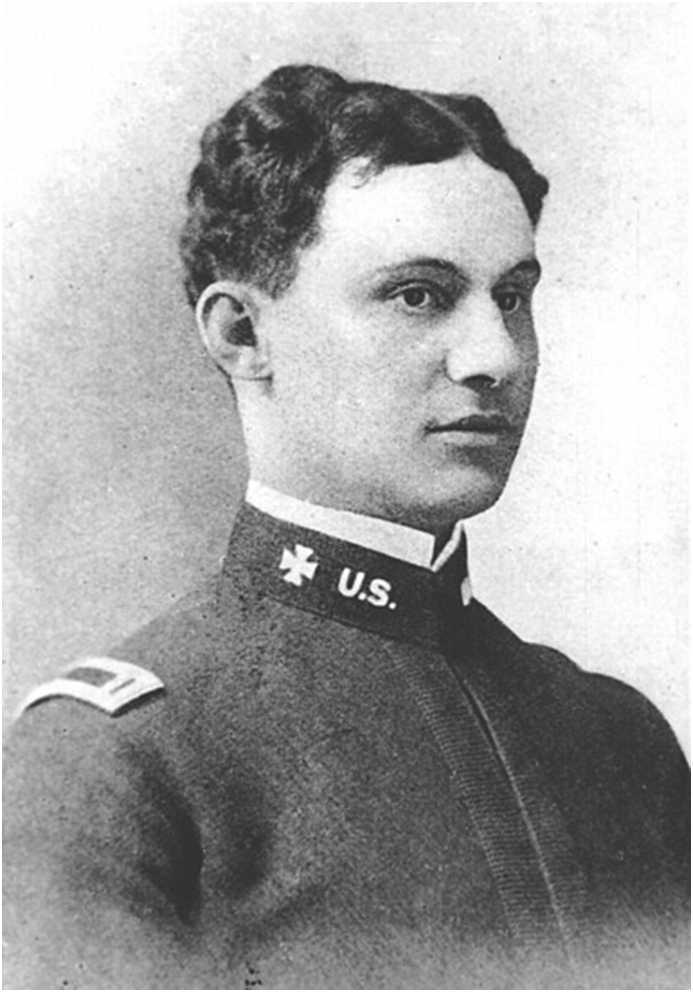
Dr H.M. Cohen. Reproduced with permission from *Br J Anaesth* 1930; **7**: 2.

**Fig 3:**
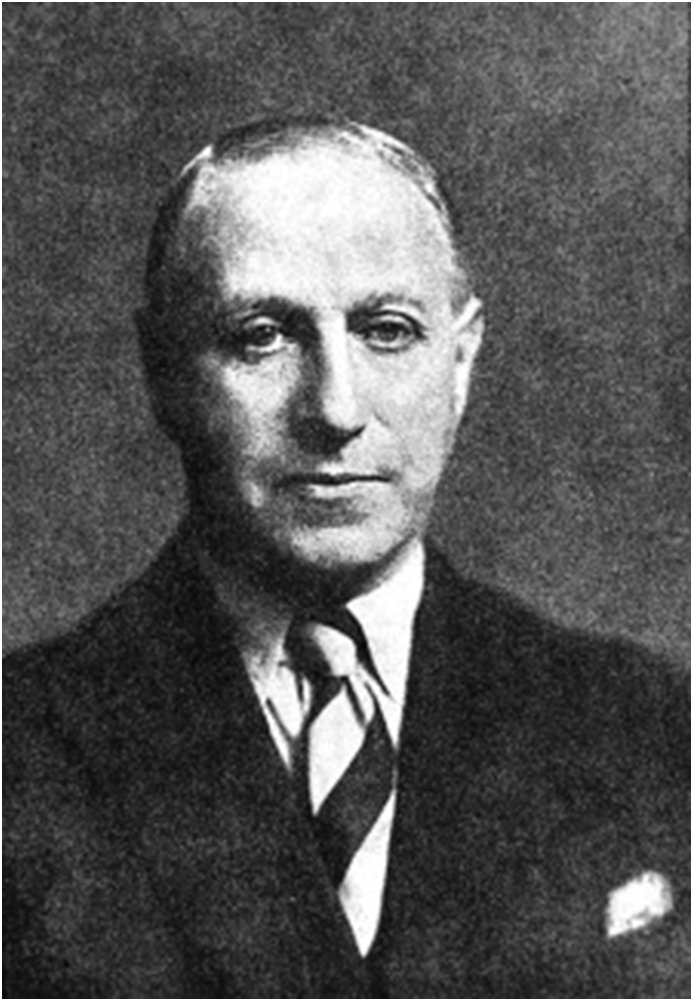
Dr J. Blomfield. Reproduced with permission from *Anaesthesia* 1949; **4**: 90.

The first few decades of the journal before World War II saw a number of hurdles, including the untimely death of the first editor H. M. Cohen (Fig. 2) and wrangling over the legal status and ownership of the journal.^7^ The long-time publisher of the journal, John Sherratt and Sons in Manchester, paid for the costs of production of the journal but kept any profit from the venture. This perhaps was one of the factors that meant that by the late 1930s, financial strain was beginning to show, and the Association of Anaesthetists of Great Britain and Ireland donated £25 to the journal in 1939 on the suggestion of Joseph Blomfield, which was gratefully received.^8^ Blomfield was both editor of the *BJA* and Honorary Secretary of the Association at this time (Fig. 3). With the outbreak of war, those journals such as the *BJA* without guaranteed subscribers faced significant challenges. Whereas journals such as *the British Medical Journal* (*BMJ*) benefitted from an uptick in members of its parent organisation, the British Medical Association (BMA), the *BJA* and others like it faced significant disruption and financial challenges. The removal of printers from the workforce as a result of conscription, rationing of paper, and disruption of in-person meetings reduced the *BJA* from four issues a year to two or one, continuing through until 1950. Although the immediate post-war years presented some opportunities for anaesthesia as a specialty, including increased numbers of practitioners with at least some degree of training, the introduction of tubocurarine as a neuromuscular blocking agent, and increased numbers of doctors delivering anaesthesia during the war, it also faced a variety of challenges. The *BJA* itself was in major trouble, both financial and organisationally. And the critical international networks of communication between other countries such as the USA and Europe and the UK were in tatters. The spread of anaesthesia knowledge through a network of shared journals and in-person communications was significantly reduced, and the regular study tours and visits between the various anaesthetic societies had been suspended. The journal limped on during these years, eventually revitalised with the beginning of the joint editorship of Professor T. Cecil Gray and Edward Falkner Hill and a return to quarterly publishing from 1950.

## The National Health Service

The Second World War also proved decisive in the establishment of the UK National Health Service (NHS). Political and social concerns about the health of the population had been growing since the beginning of the 20th century. High infant mortality rates and the poor health of the working classes drove public health initiatives, including mother and baby clinics and the distribution of vitamins, milk, and orange juice. In 1930 the BMA suggested establishing a regional hospital system and extending national insurance cover to the whole population.^9^ The Socialist Medical Association went much further by proposing a single system, administered by local government and paid for through taxation or insurance.^10^ The future of health services was a regular discussion point at the Ministry of Health through the War.^9^ The anticipation of high numbers of civilian casualties led to the establishment of the Emergency Medical Service (EMS).

The EMS brought all hospitals together on a regional basis for the first time, and the Ministry of Health commissioned the Nuffield Hospitals Trust to map hospital bed capacity, staffing, and funding. This showed that >80% of beds were in hospitals run by local authorities, with the remaining beds in voluntary hospitals managed by Boards of Governors, and revealed the unevenness in quality and distribution of health services across the regions. In 1942, the government commissioned William Beveridge, Liberal politician and social reformer, to produce recommendations for a comprehensive national health service. Beveridge set out a programme of reform that would provide state support from cradle to grave. Public opinion polls indicated strong public support, and in 1944 the government published a White Paper that set out plans for a comprehensive centrally governed health service.

The 1945 General Election produced an unexpected landslide victory for the Labour party. Prime Minister Clement Attlee appointed Aneurin Bevan as the Minister for Health, responsible for implementing plans for a health service. Bevan had spent his early life in the mining community of Tredegar in the South Wales valleys. Tredegar was unusual in having a comprehensive medical aid subscription scheme that provided care for miners and their families, including dental care. Bevan's vision for a health service that was funded through national taxation and free at the point of care was shaped by his experiences in Tredegar. There was opposition to Bevan's plans from many quarters, including local authorities who wanted to retain control of municipal hospitals, from GPs who did not want to be salaried employees, and from teaching hospitals that did not want to report into regional authorities. To create a national system Bevan was forced to compromise in order to get buy-in from the various groups. The result was a system that had inbuilt flaws from the outset. Indeed Klein argues that the 70+ yr history of the NHS can be read as a process of ‘working out’ these contradictions.^11^ But public reception of the NHS was highly positive and the take-up of GP appointments, dental, and optician services exceeded all expectations.

The new NHS brought a radical shift in the landscape of healthcare in the UK and boosted the position of anaesthetists. The new NHS required many more staff to deliver services, and anaesthesia was one of the beneficiaries of this expansion. In 1949 the NHS employed 459 anaesthetists and by 1964 this had almost doubled to 906.^12^ The post-war years also saw a shift in the position of anaesthetists. Alongside many other more established specialties, anaesthetists were successful in obtaining consultant status at the foundation of the NHS. Initially, however, it was assumed that only the three royal colleges, physicians, surgeons, and obstetricians and gynaecologists, would be included. The BMA was unwilling to assist anaesthetists in obtaining consultant status, possibly in part because of their sizeable GP membership who also administered anaesthesia. However, after the committed work by the Association of Anaesthetists to advise on anaesthetic matters during the war, the royal colleges and the government bodies were willing to accept the submissions from the Association on the inclusion of anaesthetists into the NHS at consultant status. The Royal College of Surgeons was approached about the feasibility of setting up a Faculty of Anaesthetists to meet the perceived requirements. This involved a rather rapid upgrading of the pre-existing anaesthesia Diploma to a two-part qualification that met the fellowship standard. John Gillies, editorial board member of the *BJA*, was much involved in the required negotiations between the Association of Anaesthetists and the Royal College of Surgeons. The persistence, pre-war establishment of anaesthesia qualifications, and quick response of the anaesthesia community all contributed to their eventual success in gaining consultant status. At the founding of the NHS, anaesthesia could lay claim to the essential components of a specialism: a knowledge base, specialist training, and the capacity for self-regulation.

## A new research context

The years after the end of the Second World War also saw a new research culture spreading in British clinical medicine, including in anaesthesia, overseen by the Medical Research Council (MRC). The MRC had been established initially in 1911 as part of the National Insurance Act, for research on tuberculosis, and then relaunched after the end of the Great War with a much broader remit. Dominated by Cambridge-trained physiologists, and with the help of some US funding from The Rockefeller Foundation, the MRC sought to extend the research ethos of the physiology laboratory to the clinic, not without attracting opposition from more traditional-minded practitioners.^13^ MRC activities initially, in the inter-war period, focused on promoting the basic biomedical sciences (the Ministry of Health, also newly formed in 1919, supported clinical and epidemiological research). The few clinical MRC initiatives were concentrated at London institutions.

During and after the Second World War, partly motivated by a mood favouring innovative rational solutions for old medical problems in the new NHS, such activities extended increasingly to the provinces, and the MRC took on a coordinating role. Between 1947 and 1952, more than 50 new full-time chairs in clinical subjects were established at medical schools across the country, and this substantially enhanced Britain's clinical research capacities.^14^ Before World War II, only Oxford could boast an anaesthesia department, followed in 1946 by Edinburgh, but the late 1940s and 1950s saw six new departments, and the 1960s saw a further eight formed.^15^ This development went along with a substantial reorganisation of the higher education landscape. Induced by new funding mechanisms, what used to be essentially private autonomous institutions became elements of a national university system. Regional reorganisation changed the relationship between medical schools and teaching hospitals.^16^

The *BJA* and anaesthesia community more widely were well placed to take advantage of the increase in anaesthetic research. In 1968, the Anaesthetic Research Society was formally founded, building on a more informal arrangement as a group since 1958, with the aim of furthering anaesthesia research in the UK. The formation of a potential competitor in the journal *Anaesthesia*, the Association's own journal in 1946, had prompted a number of suggestions that the two journals be merged. The last suggestion came as late as 1959, but always failed to find substantial support, and several prominent members of the anaesthesia community argued that a little ‘friendly rivalry’ between journals was in fact healthy for the specialty.^17^ As a result, the UK now had two anaesthesia journals in which to place research.

## The beginnings of clinical trials

The way research was conducted was also undergoing a shift. A new Clinical Research Board, guided and dominated by the MRC, was established to help reorganise clinical research on a national basis. Promoting the new randomised clinical trial (RCT) approach to studying the effectiveness of therapeutic interventions was part of this. The MRC considered a well-planned RCT the best way of transferring the methodological rigour of the physiology laboratory into the clinic, especially where an intervention was not obviously superior, as the methodology drew on strengths developed by MRC-funded researchers, including a strong biometric tradition.^16^ Clinicians in hospitals across the NHS were encouraged to consider research, guided by the Clinical Research Board (and thus the MRC). This included, we must assume, ambitious anaesthetists, who needed an outlet for publishing the results of their research, and the *BJA* provided such an outlet.

A special issue of the *BJA* in 1967, dedicated to clinical trials in anaesthesia, and including an MRC statement on ‘Responsibility in Investigations on Human Subjects’, provides insight into what the editors considered important in this context.^18^ The editorial framing the articles in the special issue focuses very much on practical concerns around clinical trials, suggesting that those who carry out clinical trials may acquire useful skills, but also discusses the risks of placing too much trust in statistics, warning that ‘there is a world of difference between a statistically significant and a clinically significant difference between the effects of two drugs’.^19^ There is also a reference to the thalidomide scandal in the editorial, then recent and very present in the imagination of a public increasingly sceptical about the promise of inevitable medical progress, which illustrated that dangerous side-effects can only be observed in trials when we actually look for them.

The articles in the special issue offer a variety of perspectives on issues associated with clinical trials, particularly, but not exclusively, in anaesthesia. Above all they introduce readers to issues of trial design and some of the practicalities of undertaking clinical research. Former *BJA* editor T. Cecil Gray discussed the need for clinical trials at a time when medicine was conceived of less and less as an art, and increasingly as a science. The article introduces the key principles of modern drug testing.^20^ The Leeds-based psychiatrist Max Hamilton, better known as the inventor of the Hamilton Depression Scale, contributed a programmatic article on the main premises and problems that potential organisers of clinical trials must consider.^21^^,^^22^ This included the selection of trial subjects, whether a trial had necessarily to be double-blind, the criteria for determining if effects were meaningful, and the issue of randomisation. There are also very practice-oriented articles on statistical analysis and the use of computers.^23^^,^^24^ It is evident that the editors sought to provide readers with tools and arguments for getting involved in trials themselves, and perhaps ultimately submit quality manuscripts to the *BJA*. These efforts to provide the anaesthesia community with research skills would go on to be a hallmark of *BJA* activities in the following decades. By the 1970s publications on clinical trials in anaesthesia increased in frequency and gradually began to dominate the landscape of what was considered high-quality research.

## A change of publisher and a changing journal

**Fig 4:**
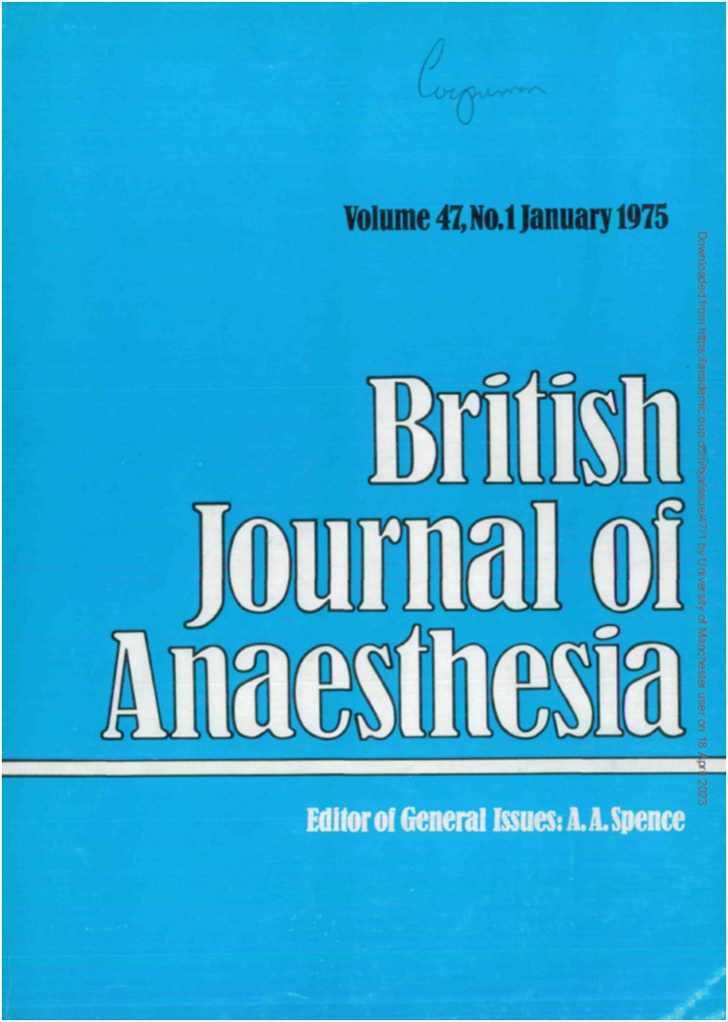
The first issue of the journal to be published by Macmillan Journals Limited, January 1975. The journal cover was redesigned to move the list of contents to the back cover, and to include a different dominant colour for each volume. Reproduced with permission from *Br J Anaesth* 1975; **47**: 1.

The year 1975 was a year of substantial change for the *BJA*, and arguably the start of the journal we see before us today. After >50 yr of publication by John Sherratt and Sons of Manchester, the *BJA* moved to publication by Macmillan (Fig. 4). This was not a universally popular decision, with both the original publishers and some board members seeing this as a betrayal of the long relationship that had seen the *BJA* through many years, including the difficulties of war and post-war rationing. However, with Macmillan agreeing to provide a significantly greater sum towards editorial expenses, and a share of the profits, the prospect of financial security for the *BJA* and a more reliable publication schedule ultimately won out.

Alongside this new arrangement, the *BJA* also began the transition to becoming a registered charity. It was no longer responsible for paying income tax, but did now need to demonstrate that it was funding charitable activities beyond the publication of a journal. As a result the *BJA* began to fund various educational activities, one of the first being a writing workshop held jointly with the *BMJ* in Glasgow in May 1979. Joint meetings were held with organisations such as the Faculty of Anaesthetists on topics such as brain ischaemia, and a hugely successful *BJA* symposium on writing and publishing papers was held at the 1988 World Congress of Anaesthesiology in Washington, DC, USA. From 1987, the journal appointed research fellows, with Jeremy Fairchild's work on the effects of calcium channel blocking drugs on cerebral ischaemia receiving the first appointment. From 1995, research grants for projects were awarded in addition to fellowships, with the intention of supporting several smaller research projects with each award. Eventually the grant-making activity was taken under the auspices of the National Institute of Academic Anaesthesia, upon its founding in 2008, and under which it continues today.

*‘The biggest change initially would be from what you might call a cottage industry, moving from Sherratt’s originally to Macmillan and then on from Macmillan. That was the big difference from being essentially a cottage industry to something much more vibrant and successful.’*

Professor William Fitch, interview with E. Shaw on July 21, 2021.

## Peer review

For many years the decision of what to publish in the *BJA* rested solely with the editors. Since at least the 1950s, however, the *BJA* did, at the editors' discretion, ask other senior anaesthetists to look at articles before publication. This was in addition to the general practice of sending particularly more complex papers to various members of the board for their thoughts, suggestions, and edits. At a board meeting on July 18, 1972, the *BJA*'s board discussed the proposal to keep a list of what they called ‘consultant editors who were not concerned with matters of policy but were simply assessors of papers’, and this proposal was approved. This was not quite the peer review process we know today, but was remarkably ahead of its time, considering the journal *Nature* introduced peer review in 1973, and *The Lancet* in 1976. Baldwin has argued convincingly that increased pressure on scientists to be accountable to funders in the wake of Cold War funding restrictions prompted scientists to devise a method that gave the appearance of a rigorous impartial review of scientific grants and publications, but still allowed scientists to maintain control of their profession.^25^ The resulting process of expert refereeing, or peer review as it began to be named, was adopted relatively slowly and only became associated with scientific legitimacy in the latter decades of the 20th century. Through the late 1970s, the process of peer review was refined at the *BJA*. There was already, however, recognition of the intense pressure this placed on journals. The editor Professor Alastair Spence in 1981 said ‘There are signs, however, that this function of journals may become overloaded, and it is time to examine alternatives’.^26^ Despite these types of critiques of peer review across the decades, peer review has been established as an important cornerstone of the scientific publishing process, and the *BJA*'s commitment to rapid and constructive peer review has become an important part of its identity.

*‘My first contact with the journal was as an author, because I had an opportunity to publish in the journal one of my most important papers, even when I was at the early stage of my research career. And I was impressed by the quality of the reviews that I received from reviewers selected by the editor. And I realised that when you have the opportunity to send a paper, to submit a paper to a journal like this, whatever the decision will be, your paper will turn better at the end of the editorial process.’*

Andrea Cortegiani, interview with E. Shaw on November 4, 2021.

## Continuing innovation

In 1990, the *BJA* became the official journal of the College of Anaesthetists (later the Royal College) while maintaining editorial and financial independence. The following years saw an increasing rate of technological advancement, initially beginning with the introduction of manuscript handling on computer disks. The collaboration between the *BJA* and *Anesthesiology, Anesthesia & Analgesia*, and the *Canadian Journal of Anaesthesia* brought five years of those journal's issues together on CD-ROM (1991–1995). The Electronic Anaesthesiology Library, or TEAL, released in 1996, was searchable, a major innovation. The introduction of the software package Manuscript Central in 1999 meant that instead of vast quantities of manuscripts being posted into the journal, between referees and editors across the country and frequently abroad, instead electronic submission and review was possible. This went some way to dealing with the increasing number of manuscripts submitted to the *BJA*, and significantly decreased the time it took to peer review and edit papers, something attractive both to potential authors and the editorial team.

*‘That’s been the major step in certainly the last 25 years, of moving into the electronic age. And we were one of the first, certainly one of the first anaesthetic journals, in the world to do that. I think some people thought I was a bit mad. But it was definitely the right way to go, all journals do it now.’*

Professor Jennie Hunter, interview with E. Shaw on November 30, 2020.

Alongside this hugely successful innovation was an attempt to take the journal online, less successful but hugely ahead of its time. Significantly reduced printing and postage costs were appealing to some members of the board. In order to draw attention to the internet version of the *BJA*, it was decided that an abbreviated paper version called *BJA Concise* would be produced. This would contain abstracts of the online papers, in an effort to direct people to the online version.

*‘I did something which was described as being very brave by other editors, we did indeed produce an internet only version of the BJA, but we decided that we should draw this to the attention of readers by producing the BJA Concise. But after six months we abandoned it and went back to paper and internet versions of the BJA. The main reason was that the number of submissions from particularly nonclinical scientists dropped like a stone. I wrote an editorial at the end of this experiment, when we’d abandoned the BJA Concise, to explain why we had tried this experiment and why we had abandoned it.”*

Professor Graham Smith, interview with E. Shaw on September 16, 2021.

In spite of the short-lived nature of *BJA Concise*, with the benefit of hindsight, we can all see that online publishing has radically altered the academic publishing industry. Now the almost entirely online nature of information exchange has transformed both the day-to-day work of anaesthetists and the global responses to major events.

## COVID-19

**Fig 5:**
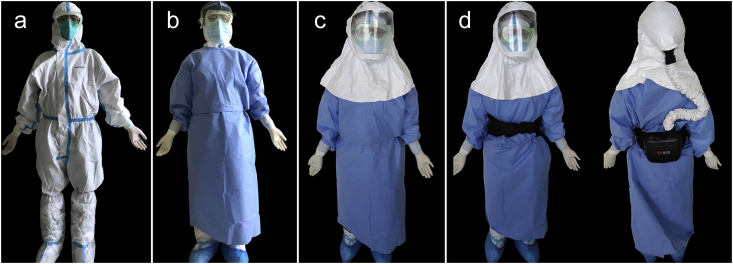
Layers of personal protective equipment to provide protection for medics working with patients with COVID-19. Reproduced with permission from *Br J Anaesth* 2020; **125**: 1.

At the start of 2020, reports of a novel coronavirus causing respiratory illness in China dominated the news cycle. By the middle of February, the WHO had declared the outbreak a Public Health Emergency of International Concern (PHEIC), naming the illness caused by this novel coronavirus ‘COVID-19’. The impact of COVID-19 on the journal was immense. The *BJA* editorial team approached a team of anaesthetists who had experience of managing other outbreaks to write an editorial for the journal with their insights on managing a highly contagious respiratory disease. Peng and colleagues wrote an editorial outlining the outbreak of COVID-19 so far and communicating lessons for anaesthetists, already anticipating the risk of significant levels of infection amongst healthcare workers (Fig. 5).^27^ This was one of the first articles to address COVID-19 in any anaesthesia journal. The article was, by a significant margin, the most cited article from the BJA from 2020. It was the first in a major effort from the editors and peer reviewers to review submissions and expedite publication via advance access. In 2020, the *BJA* published 500 more articles than it had in the preceding year, with the overwhelming majority of these being related to COVID-19. The journal identified that it had a responsibility to its readers and the community more widely to publish whatever it could, as a gesture of solidarity, support, and a provision of guidance.

*‘We published everything to do with COVID-19 free of charge so that it was accessible to anyone. And for us, that was our service to the community, to our colleagues, to the field and to people of the world. Any information potentially valuable to clinicians taking care of patients with COVID-19 or to scientists doing research on COVID-19, we made freely available, accessible to anybody.’*

Professor Hugh Hemmings, *BJA* editor-in-chief, interview with E. Shaw on March 22, 2021.

## Conclusions

The action taken by the *BJA* team to meet the challenge of the COVID-19 pandemic shows how the *BJA* has cultivated a mission of providing a service to the anaesthesia community. In the early days, the *BJA*'s proactive stance focused more on advocating for change in working conditions and pay. With the transformation of the nature of anaesthesia research in recent decades, the focus has now shifted to promoting high-quality anaesthesia research, through publishing, funding and educating the anaesthesia community. Radical shifts in the academic publishing industry, the UK health system, and the organisation of medical research have required the *BJA* to change and adapt in order to survive an often turbulent publishing landscape. The recent decision to move to electronic only publishing is yet one more transformation in a long line of shape shifting to meet the challenges of the times. In many ways, anaesthetic practice is now unrecognisable from the often haphazard context of anaesthesia in the early 20th century. The introduction of the NHS and significant developments in anaesthesia practice have transformed a tiny community of enthusiasts into the largest hospital specialty in the UK. The expansion of research funding, organisation, and design has propelled anaesthesia research from dangerous self-experimentation, such as the death of Sidney Rawson Wilson while experimenting with nitrous oxide in 1927, to huge multicentre RCTs with budgets in the many millions of pounds. That the *British Journal of Anaesthesia* has managed to not only survive, but thrive throughout these significant changes shows the value of reinvention, and the importance of taking risks to meet changing times. Although not always immediately successful or valued at the time, the bold decisions of many editors and boards of the Journal have contributed to its ability to celebrate its centenary in 2023. All that can be guaranteed about its future is that many more challenges await.

## Authors’ contributions

Overall conception and writing of the paper based on research on the *BJA*: ES.

Drafting and revising, overall approval before submission: all authors.

Section on the history of the NHS: SS.

Section on the history of the MRC: CT.

## Declaration of interest

The authors declare that they have no conflicts of interest.

## Funding

This work derives from the PhD studies of Eleanor Shaw supported by a grant from the *British Journal of Anaesthesia* (WKR0-2018-0070) through the National Institute of Academic Anaesthesia.
